# The use of molecular genetics in environmental harm studies: eDNA as a method for ecological damage in the ecocriminological analysis

**DOI:** 10.12688/openreseurope.21126.1

**Published:** 2025-08-18

**Authors:** Esteban Morelle-Hungría

**Affiliations:** 1 Senior Lecturer in Criminology. Public Law Department, University Jaume I, Castellón de la Plana, Valencian Community, Spain

**Keywords:** eDNA, environmental harm, molecular genetics, ecological damage, green criminology

## Abstract

This research aims to explore the potential of molecular genetics in general, but in particular the use of environmental DNA (eDNA), as an innovative tool to complement the assessment and valuation of environmental harm from the perspective of green criminology or ecocriminology. In the face of the planetary crisis and the possible collapse of ecosystems, it proposes to overcome the limitations detected in traditional criminal law by adopting an ecocentric approach. This implies recognising the intrinsic value of nature and the need to commit to ecological justice. To this end, the use of eDNA makes it possible to visualise the biological and ecological transformations induced by pollutants, even when they are not visible to the naked eye. This provides objective, and even quantified, data on damage such as biodiversity loss, community alterations or possible genotoxic effects. It is presented as a complement to support preventive and restorative interventions in environmental crimes. Through dialogue between science, law and ethics, an integrated paradigm of environmental harm analysis is advocated, in which molecular genetics reinforces the ability to detect, understand and respond to ecological damage. The convergence between green criminology, eDNA and ecological justice allows us to refocus institutional responses towards restoring the integrity of ecosystems and defending the rights of nature.

## Plain Language Summary

This article explores how new scientific tools can help us detect and understand environmental damage more effectively. It focuses on a method called environmental DNA, or eDNA, which allows scientists to collect tiny genetic traces that animals and plants leave behind in water, soil, or air. By analyzing these traces, we can know what species are (or were) present in an ecosystem, even if they are no longer visible. This is very useful when trying to identify pollution or the disappearance of certain species due to human activity. The article also connects this scientific method with green criminology, which studies how human actions harm the environment—even when those actions are legal. It argues that we need to recognize nature as something that has value in itself, not just because it benefits humans. By combining science, law, and ethics, the study suggests new ways to identify who is responsible for ecological damage and how we can restore the natural balance and protect the rights of nature.

## Introduction

We are witnessing a planetary crisis in which the current risks and problems facing all species are unprecedented ( Marteache Solans, 2024). This situation stems, among other things, from human activity linked to a model mired in excessive consumerism, which implicitly involves the accelerated loss of natural resources. The planet in general, but particularly biodiversity and ecosystems, are the most affected, and therefore planetary boundaries represent a new perspective for shifting towards a comprehensive and holistic model of integral protection of the planetary balance, given that “one health” is being affected ( Alonso & South, 2025; Morelle-Hungría, 2022). The scientific literature in this area paints a devastating picture. For example, around one million species may be at risk of extinction in the coming decades, a milestone in history as this number has never before been quantified ( IPBES, 2019). To this must be added the consequences and impacts derived from global problems such as global warming ( IPCC, 2021). This highlights the level of anthropogenic pressure exerted on the planet's ecological systems ( IPCC, 2021). In this context, traditional criminology – focused on conventional crimes and an anthropocentric approach – is ineffective in understanding and addressing the complexity of ecological damage ( South, 2014). Green criminology is a perspective of great interest because, among other things, it allows criminology to focus on environmental harm as an object of study. For example, reaching extremes never seen before many of the actions that are harmful or risky to nature are classified as lawful activities or even carried out by the administration itself ( Lynch & Stretesky, 2014; Morelle-Hungría, 2020).

This criminological perspective implies essential transformations are needed for the comprehensive analysis of ecological damage. On the one hand, it broadens and redesigns the notion of crime by emphasising the concept of harm. Beyond the formal violation of criminal laws, it is argued that acts or omissions that cause negative damage to ecosystems, fauna, flora and even ecological processes should be considered crimes ( Brisman, 2014; Lynch & Stretesky, 2014). Similarly, it broadens the concept of victim to include non-human species, the environment and even the entire planet as subjects worthy of protection ( Brisman, 2014; Morelle-Hungría, 2023). Consequently, the category of environmental offender is no longer limited to the tangible individual who dumps waste or poaches but also encompasses structural actors and factors—such as corporations, states and even global economic dynamics—ultimately responsible for ecological collapse ( Brisman, 2014). Thanks to the green perspective, criminology provides us with a critical and holistic view that allows us to question the political, economic, and cultural roots of ecological degradation. Advances in molecular biology have opened up a new avenue for improving our comprehensive understanding of the damage caused. With the incorporation of tools and methods such as environmental DNA (eDNA) analysis, it is possible to monitor the health of ecosystems. This allows us to identify the presence or absence of species in the ecosystem, opening up new lines of research ( Bunce & Freeth, 2022). This mechanism also allows us to identify a ‘biological footprint’ that reveals the composition of a given habitat without resorting to invasive forms of evidence collection ( Bunce & Freeth, 2022). These techniques have been used in other fields and areas of knowledge, such as ecology and even forest management, providing a more holistic view for analysing the biological networks that sustain ecosystems. For green criminology, this can be useful for highlighting ecological transformations, even the most subtle ones, produced by certain human activities.

Another key conceptual axis in this analysis is ecological justice. Derived from an ecocentric worldview, ecological justice proposes that the moral community should extend beyond humanity to include non-human nature, recognising the intrinsic value of ecosystems and demanding their protection as a right in their own right ( White, 2018). We cannot ignore the fact that without ecosystems and biodiversity, life cannot be guaranteed, let alone human rights, including the right to a healthy environment, which is why this argument alone would be sufficient to grant fundamental and non-instrumental legal value ( Borrás-Pentinat, 2024). In order to achieve this recognition, most countries should implement the recognition of the rights of nature. In this regard, we agree with Borrás-Pentinat (2024, p. 235) when he states that this requires the intervention of both individual states and the European Union itself.

Unlike traditional environmental justice, which tends to focus on the equitable distribution of environmental harms among human groups, ecological justice focuses on the well-being of the natural environment itself and all the beings that comprise it, advocating for the repair of ecological damage itself and the restoration of lost balance ( Morelle-Hungría & Serra-Palao, 2024; Sollund, 2022). Within the framework of green criminology, ecological justice serves as a guiding principle that informs both our understanding of the problem (who or what is harmed when we degrade nature?) and our responses to it (how can we remedy that harm in a comprehensive way?). This approach rejects individualistic bioethical views—for example, debates focused exclusively on the rights of the human being involved—and transcends the traditional punitive paradigm, emphasising the restoration of ecological systems and the prevention of further harm as a measure of justice ( Di Ronco & South, 2025; Morelle-Hungría & Serra-Palao, 2024). This research develops a theoretical and conceptual analysis of ecological damage from the perspective of green criminology. We do so for the first time, integrating contributions from molecular genetics and intersecting with the notion of ecological justice. Next, we will focus on the theoretical framework, where we will first describe the foundations of green criminology and ecological justice. Subsequently, we will show how molecular genetics can contribute to broadening our understanding and analysis of environmental harm caused by human activities. In addition, we will explore the intersection between green criminology and molecular genetics in order to characterize ecological transformations, offering a systematic view of the damage caused by different activities.

## Theoretical framework

### Green criminology and environmental harm

The beginning of this criminological perspective, described as green, dates back to the early 1990s, in response to concerns about the serious environmental consequences of human conflicts, especially given that the approaches implemented were inadequate to deal with these situations ( Marteache Solans, 2024). It is recognized as the beginning of green criminology, proposing an ecologization of criminology as a new paradigm focused on human acts that cause damage to the environment, questioning the traditional indifference of criminology itself towards crimes without human victims ( Lynch, 1990) while South (1998: 226) suggested that “Green issues open up a wide range of possibilities for interdisciplinary work, both within the social sciences and with disciplines in the natural sciences”. This trend gained momentum in the following decades, consolidating itself theoretically through fundamental works (e.g. Brisman & South, 2013; Lynch & Stretesky, 2014; White & Heckenberg, 2014) that laid the foundations for a "non-anthropocentric" criminology ( South, 2014).

An essential starting point for green criminology is the critique of the anthropocentrism that dominates criminal law and traditional criminology ( García Ruiz & Morelle-Hungría, 2023). According to South (2014), the classic utilitarian view conceives of nature merely as a resource at the service of human interests, focusing on environmental harm only to the extent that it directly affects people or their property. This leaves a wide spectrum of systematic ecological aggression outside the criminological focus, especially where harm falls mainly on animals, forests, rivers or other natural elements without an immediate link to human victims ( South, 2014). Green criminology breaks with this limitation by asserting that the environment itself can be the victim of injustice and harm, and therefore the subject of criminological attention ( Brisman, 2014). This entails broadening the definition of "crime" beyond legal transgression to include any human action or omission that causes significant ecological harm, even if such action is legal under current regulations ( Vegh Weis, 2024). This approach allows for practices that also cause harm but are socially or legally tolerated – such as industrial discharges within permitted limits, "legal" deforestation, overfishing, carbon emissions that comply with lax regulations, etc. – but which cumulatively degrade the biosphere to such an extent that they may exceed activities considered illegal, including ( Colás Turégano & Morelle-Hungría, 2021; García Ruiz, 2022; García Ruiz & Morelle-Hungría, 2023; Morelle-Hungría, 2020). The central idea is that the parameter for considering something criminologically relevant is the harm caused, not just its legal status ( Lynch, 1990; Lynch & Stretesky, 2014).

Green criminology proposes analysing phenomena such as chronic pollution, biodiversity loss and climate change as forms of environmental crime or victimisation, even if they do not always fit into typical criminal categories. Lynch and Stretesky (2014) advocate a "green revolution" in criminology where "green harms" – a term that encompasses damage to ecosystems and animals – are taken as seriously as traditional crimes against people or property. This also implies questioning the power structures that allow such harms to occur. Many environmental crimes are not the work of isolated individuals but are linked to structural dynamics: economic development models, extractive policies, mass consumption and corporate cultures focused on immediate profit over sustainability ( South, 2014). Green criminology, influenced by critical criminology and the "treadmill of production" theory of environmental sociology, examines how global capitalism and the pursuit of perpetual economic growth contribute to the overexploitation of nature, creating systemic "environmental criminogens" ( Lynch & Stretesky, 2014). It identifies macro-structural factors as fundamental causes of ecological damage, such as polluting transnational corporations and international supply chains, among others ( South, 2017). This alone broadens the traditional focus on individual criminals to include white-collar crime and corporate crimes related to the environment ( Lynch *et al.*, 2020).

In this context, the green perspective is shaped by various theoretical frameworks. On the one hand, critical criminology and Marxist theories are integrated to understand the political and economic dimensions of the problem. On the other hand, it incorporates expanded victimology by considering a broader view of the victims of this type of crime in order to protect vulnerable communities and even nature itself ( Marteache Solans, 2024). In turn, connections with other disciplines further removed from criminology are taken into account: ecology, ethics, international law, and environmental justice studies. All of this shapes a multi-level, interdisciplinary approach aimed at comprehensively understanding environmental harm, its causes, and its consequences.

A relevant conceptual distinction within green criminology is that of "environmental justice" versus "ecological justice". Traditional environmental justice deals with the equitable distribution of environmental burdens among different human groups (e.g., ethnic minorities, poor communities disproportionately affected by pollution), while ecological justice—as we will see in detail below—extends the sphere of justice to nature itself. Both strands inform green criminology: on the one hand, there is concern about the unequal impacts of environmental degradation on vulnerable human populations (e.g., indigenous peoples affected by extractivism); on the other hand, it is recognised that the devastation of species and habitats poses a justice problem that transcends anthropocentrism ( White, 2013).

Green criminology provides an expanded theoretical and conceptual framework for analysing environmental harm: it defines such damage as a legitimate object of criminological study, questions legal boundaries and proposes an ecocentric view in which protecting ecological integrity becomes a central imperative ( Brisman, 2014). It is not just a matter of cataloguing new "environmental crimes", but of rethinking what we understand by crime, victim and justice in the Anthropocene era, where human actions threaten the biophysical stability of the planet ( South, 2014). Having established this conceptual framework from the perspective of green criminology, we will now explore how environmental molecular genetics can complement and enhance this perspective by providing scientific evidence of changes and damage to ecosystems.

### Environmental molecular genetics: making ecological damage visible

As we have already mentioned, molecular genetics applied to the environment allows us to integrate a set of techniques and approaches that use DNA or other genetic markers to study ecological components and processes. Environmental DNA (eDNA) analysis has become an effective technique for monitoring biodiversity and tracking ecological changes. eDNA is the genetic material that organisms leave behind or shed in their environment, such as skin cells, fences, hair, pollen, spores, etc. By collecting samples of water, soil or air and extracting this residual DNA, it is possible to identify which species have been present in a given location, even without observing them directly ( Lewis *et al.*, 2024). This represents a paradigm shift in environmental monitoring: it is no longer essential to have sight of or capture specimens; the "genetic signature" they leave behind is enough to reveal their presence.

The proven uses of eDNA in environmental science are numerous. There are already promising results, for example, for conducting biodiversity inventories in certain ecosystems: rivers, lakes, and even oceans, detecting species that are difficult to sample using other methods. They can also be used to monitor protected or endangered species, for example, by searching for their DNA in the water to confirm whether or not they inhabit that area. In addition, it has proved very positive for the early detection of invasive alien species and pathogens before they proliferate uncontrollably. Furthermore, the use of this technique allows studies to be carried out at different time scales to monitor medium- and long-term changes in the species inhabiting a given ecosystem.

These types of techniques have solid scientific and academic backing. Authors such as Lewis *et al.* (2024) point out that the scientific community has gained confidence in eDNA methods as their effectiveness has been confirmed in various contexts of conservation, wildlife management and climate change studies. Such is the degree of consolidation and growing confidence in this tool that its incorporation into forensic investigations of wildlife and other environmental crimes is now being explored, given its ability to provide robust biological evidence ( Lewis *et al.*, 2024). However, it is important to note that in this analysis we will not focus on the use of eDNA as evidence in legal proceedings, but rather on its value in understanding and highlighting ecological damage from a systemic perspective that can help us increase the effectiveness of protection mechanisms. As Fernández-Hernández (2024) points out, legal systems must be effective in ensuring that the legislative techniques used to incorporate new types of crimes related to nature are updated and refined.

How, then, does molecular genetics contribute to making environmental harm visible and characterising it? Firstly, by detecting biodiversity loss. Many environmental impacts manifest themselves not so much by the presence of something, but by the absence or decline of sensitive species. For example, certain aquatic insects may disappear from a river after it has been polluted with agrochemicals, or the diversity of soil microbes may collapse due to toxic spills. Traditionally, recognising these losses required lengthy field sampling and expert taxonomic knowledge. With this type of technique, it would be enough, for example, to analyse water or soil samples to determine whether there are signs of the disappearance or reduction of genetic sequences of certain species, thus allowing us to establish an early warning signal ( Bunce & Freeth, 2022). In fact, eDNA offers much greater temporal and spatial resolution than traditional methods, allowing for near real-time monitoring of biota. This issue is interesting even for environmental criminal law itself or for green criminology, as many forms of environmental harm are gradual and cumulative; the ability to detect them at an early stage facilitates the assignment of responsibility and the adoption of preventive measures before major collapses occur.

Secondly, molecular genetics allows us to characterize changes in biological communities induced by external and internal disturbances. Not all pollutants kill life immediately; some generate ecological subtleties, such as changes in species composition (some proliferate, others decline). For example, chronic discharge of nutrients or heavy metals into an ecosystem may not exterminate all life, but it may favour opportunistic organisms (algae, tolerant bacteria) to the detriment of more sensitive species, profoundly altering the trophic web. These transformations can be detected by eDNA metabarcoding, which identifies the sequence of multiple species simultaneously and quantifies their relative abundances. This makes it possible to profile "genetic signatures" of pollution: characteristic patterns in environmental DNA that reveal, for example, the dominant presence of microbes’ indicative of organic pollution, the absence of certain invertebrate bioindicators of clean water, or the anomalous abundance of metal resistance genes in the microbiota (a sign of exposure to heavy metals). Such findings provide an objective description of the damage: they not only confirm that there was an impact, but also how the local ecology has been altered.

An illustrative case comes from the analysis of marine environments affected by authorised human activities. Morelle-Hungría and Serra-Palao (2024) studied the effect of brine discharged by desalination plants on a Mediterranean seagrass bed, *Posidonia oceanica,* a key species protected both at Community level and as a priority habitat. This study highlights the ecological damage caused by this legal discharge and emphasises the need to address it through ecological justice theory with mitigation and adaptation techniques to improve the effectiveness and efficiency of the facility ( Morelle-Hungría & Serra-Palao, 2024). In fact, the authors propose solutions based on environmental restorative justice, seeking to repair the affected ecosystem (e.g., mitigation of the discharge, restoration of the seagrass meadow) rather than simply sanctioning the plant operators ( Morelle-Hungría & Serra-Palao, 2024). With the implementation of eDNA techniques in this study, it would be possible to further analyse the ecological impact caused by these physicochemical changes in the affected ecosystems, as well as the timelines to show the cumulative capacity of the impact generated. In addition, it would be possible to determine which species may have been affected by this type of discharge, issues that would benefit and optimise the research carried out.

Another contribution of molecular genetics is the detection of genotoxic effects in organisms exposed to pollutants. Genetic biomarkers indicate DNA damage in individuals or communities (e.g., high mutation rates, chromosomal aberrations, or changes in gene expression associated with chemical stress) ( Bickham *et al.*, 2000). Although this approach focuses more on individuals than on communities, its ecological implications are clear: a fish population slowly poisoned by mercury may not exhibit sudden mortality, but it will show DNA damage and reduced reproductive success, foreshadowing a population collapse. Environmental forensic genetics can therefore reveal this "invisible damage" by measuring, for example, the frequency of micronuclei in blood cells of wild animals from contaminated areas (an indicator of sublethal genetic damage). A promising area is the integration of genomic data to understand long-term systemic effects. Thanks to mass sequencing and bioinformatics, it would be possible to analyse entire communities of microbes, plants and animals and how they interact. For example, sequencing the metagenome of a soil can reveal the loss of key metabolic pathways (such as nitrogen fixation) due to the local extinction of certain bacteria following contamination. This level of detail reinforces a systemic understanding of damage: it is not just that this or that species is missing, but that essential ecological functions have been impaired. For green criminology, this is of great importance as it provides an ecosystemic view of environmental harm, which is perceived not only as isolated events but as disturbances to an interconnected system.

Molecular genetics techniques make it possible to assess and quantify damage and provide a valuable complement to the comprehensive assessment of damage with tangible elements. In addition, it facilitates the prevention of environmental harm, as it allows evidence to be gathered on the presence of certain pollutants or their effects, which would improve proactive environmental protection mechanisms. Recent studies have identified, for example, the presence of human DNA in water samples, opening up new possibilities for the use of this technology ( Goray *et al.*, 2024). These techniques can improve the assessment and even quantification of damage, providing a valuable mechanism for the comprehensive evaluation of damage caused by human activities. From a green criminology perspective, these types of tools could be used as a deterrent within general prevention mechanisms, as criminals would know that the damage they cause leaves a genetic trail and can be traced, reducing their sense of impunity. As Bunce and Freeth (2022) point out, eDNA has the potential to improve the effectiveness of protection mechanisms by increasing the visibility and understanding of the ecological consequences of our actions.

### Ecological and restorative justice as guiding principles

Within the normative and ethical framework of green criminology, the concept of ecological justice occupies a central place. As mentioned above, ecological justice is based on the idea that nature has intrinsic value and that human beings have an obligation to respect ecological limits and the rights of other species and ecosystems to exist and thrive. This concept complements the notion of environmental justice—which typically focuses on unjust relationships between human groups and the environment—and expands it towards a biocentric or ecocentric ethic ( White, 2018).

In practical terms, embracing ecological justice implies three things for the analysis of environmental harm: recognition, responsibility and reparation.

Recognition means admitting that damage to the natural environment is in itself a wrong that must be recognised by our justice institutions, regardless of whether it can be translated into terms of human injury. For example, the disappearance of a coral reef or the extinction of a species has moral and legal relevance beyond any associated economic loss or impact on human well-being. In green criminology, this recognition is reflected in the proposal of concepts such as ecocide, understood as human acts that cause massive damage to the environment and deserve international criminal punishment in the same way as crimes against humanity ( Agnew, 2020; Higgins *et al.*, 2013). Although the debate on ecocide belongs to the field of emerging criminal law and we will not delve into its legal admissibility here, the mere discussion of it reflects a change in sensitivity: it is beginning to be accepted that devastating entire ecosystems is a fundamental injustice that must be prevented ( García Ruiz *et al.*, 2022).

Responsibility in terms of ecological justice means broadening the range of those obliged to answer for environmental harm. It is not limited to the individual who directly polluted a river but includes collective and generational responsibility. This links to the idea of intergenerational justice (leaving a healthy environment for future generations) and the responsibility of corporations and states ( Ruggiero & South, 2010). For example, industrialised countries and large polluting companies have a disproportionate responsibility for the global ecological crisis, and ecological justice – or an international law of ecocide - would demand reparations or compensation from them to the countries and communities most affected by environmental degradation ( Higgins *et al.*, 2013; Lynch *et al.*, 2013). It has been estimated that 75% of environmental harm to nature is caused by legal entities ( Cuerda Arnau, 2023). From this perspective, environmental impunity stems not only from a failure to enforce laws, but also from a world order that allows certain actors to profit while transferring environmental costs to third parties (often poor populations or nature itself). Green criminology analyses these power imbalances and attempts to highlight them as structural injustices.

Reparation implies that, in the face of proven environmental harm, the priority response of justice must be to restore, as far as possible, the ecological balance that has been disrupted. This connects with the philosophy of green restorative justice. Rather than focusing exclusively on punishing the offender, restorative justice seeks to involve all affected parties (including the community and even, metaphorically, the environmental victim represented by scientists or environmental advocates) in agreeing on actions to repair the damage and prevent its recurrence. For example, if a company dumped waste into a lake resulting in the elimination of species, an ecological-restorative justice approach would propose: 1) requiring the company to finance the cleanup and recovery of the lake (replanting species, reintroducing fauna, decontaminating sediments); 2) ensuring the participation of the local community in the supervision of such restoration; and 3) encouraging a change in the company's practices so that the event does not happen again, perhaps through technological changes or permanent monitoring. In many current legal systems, restorative measures are secondary to criminal or administrative sanctions; green criminology advocates reversing this priority, placing the healing of the ecosystem at the centre of the response to ecological crime ( García Ruiz & Morelle-Hungría, 2023; Varona, 2022).

The concept of ecological justice, therefore, reorients the very purpose of social reaction to environmental harm. It is no longer just a matter of punishing the violation of a rule (which, in traditional environmental crimes, often boils down to fines that companies consider "costs of doing business"), but of restoring ecological integrity and the harmonious relationship between humans and nature ( García Ruiz & Morelle-Hungria, 2023). This also ties in with acknowledgment of and need to preserve the knowledge of Indigenous peoples and Indigenous worldviews that conceive of human beings as part of a greater web of life to which respect and obligations are owed ( Marteache Solans, 2024). In contemporary green criminology, especially from global Southern perspectives, this notion is captured through the idea of combating "ecological discrimination"—the marginalisation of nature in our considerations of justice ( Goyes *et al.*, 2021; Goyes, 2023) —and promoting rights of nature in constitutions and laws.

By incorporating ecological justice as a guiding principle, our analysis of environmental harm is enriched in several ways. First, it offers an evaluative criterion for judging the seriousness of harm: for example, the disappearance of an entire species or the destruction of a unique ecosystem should be considered a major wrong, regardless of its economic value, because it means the irretrievable loss of an irreplaceable component of the Earth (an attack on "ecological integrity," this approach would say). Second, it clarifies the ultimate objective of criminal intervention: the goal is to safeguard and, when necessary, restore the balance of life systems, rather than satisfy an abstract desire for retribution. This does not exclude sanctions but makes them subordinate to the restorative end. Third, ecological justice strengthens the argument in favour of policies to prevent environmental harm: if we recognise the inherent rights or interests of nature, the state and society would have a proactive obligation to prevent their impairment (precautionary principle), not just to react once the damage has been done.

In short, ecological justice provides the ethical and legal compass for green criminology, and it is argued here that it could be usefully complemented by the technical tools of molecular genetics and an interdisciplinary understanding of harm. While genetics shows us what happens in an ecosystem at the micro and macro levels (genetic losses, community alterations), ecological justice guides us on why that damage is important and how we should respond collectively. In the next section, we analyse how the convergence of these elements—green criminology, genetic science, and ecological justice—enables us to develop a more comprehensive model for analysing environmental harm, making previously ignored impacts visible and promoting solutions aimed at restoration and prevention.

## Discussion: Integrating science and criminology to highlight environmental harm

The combination of green criminology and environmental molecular genetics, under the umbrella of ecological justice, offers us an innovative and powerful approach to understanding environmental harm in an ecological and systemic way. In this discussion, we will address three key dimensions of this integration: (1) making environmental harm visible – how molecular evidence can bring to light previously hidden or disputed damage; (2) comprehensive and systemic understanding – how joint criminological and scientific analysis reveals the interconnected complexity of environmental issues, from genetic to social levels; and (3) implications for ecological justice – how this approach can strengthen the application of principles of restorative justice and prevention.

### Making the invisible visible: molecular evidence of ecological damage

One of the historical obstacles to the legal protection of the environment, both at the criminal and administrative levels, has been the 'invisibility' or diffuse nature of much damage. Starting with the most obvious, the criminal sphere, where unlike a common crime (e.g., theft or assault) the consequence is immediate and tangible, ecological crimes often operate silently, gradually, and in a dispersed manner. For example, how can one prove that a decline in fish populations in a river is due to discharges from a factory located upstream? Or how can one demonstrate that a permitted practice, such as agriculture using agrochemicals, is causing the loss of pollinators in a region?

Thanks to eDNA and other genetic techniques, it is now possible to establish stronger causal links between polluting activities and ecological changes. If eDNA analysis in an ecosystem adjacent to an industrial area shows that sequences corresponding to certain sensitive species (amphibians, insects) have disappeared, coinciding with the presence of toxic compounds in the water, this genetic-environmental correlation can reinforce evidence of anthropogenic damage ( Lewis *et al.*, 2024). Similarly, genetic profiles can reveal specific signatures of pollution: for example, the predominant presence of bacterial hydrocarbon degradation genes in marine sediment suggests oil pollution, or the detection of harmful algae DNA in a water column may indicate eutrophication due to excess nutrients. These types of molecular markers are used to configure bioindicators that provide quantitative and objective data that are difficult to ignore. As Lewis *et al.* (2024) point out, incorporating eDNA data into environmental research provides a level of scientific confidence that could make these tests more acceptable to various forums, including decision-making and public policy development.

### Systemic and interdisciplinary understanding of environmental harm

The integration of molecular genetics tools into the criminological analysis of environmental harm not only provides scientifically sound empirical data, but also enriches our qualitative and quantitative understanding of the damage. By adopting this approach, we can perceive the problem at multiple scales, from the microscopic/genetic to the macroecological and social, and understand their interrelationships. This is consistent with a truly systemic view, in line with the complexity of today's environmental challenges.

A notable aspect is the convergence of languages and concepts between the natural sciences and the social sciences around the environment. For example, in ecology, we talk about the 'resilience' of ecosystems to refer to their ability to absorb disturbances and recover. In green criminology, we can adopt this concept to assess how resilient an environment is to certain human activities: environmental genetics can indicate, through genetic variability or functional redundancy (several species fulfilling similar roles), how vulnerable or resilient an ecosystem is to an impact. A forest with high genetic diversity is likely to tolerate moderate logging better than a genetically degraded forest. If genomic analyses show that the genetic variability of key populations has been eroded (e.g., all mangrove trees are virtually clones due to historical deforestation), this warns that the ecosystem has lost its capacity to respond and that the next impact could be catastrophic. Green criminology, informed by this data, will know to insist on urgent protective measures in such a case. We see, then, how a biological indicator translates into a criminological priority.

Similarly, the notions of "trophic cascades" or "cascading effects" from ecology can shed light on the criminological consideration of certain types of damage. The disappearance of a predator species through illegal poaching can trigger an uncontrolled proliferation of herbivores, which in turn decimate vegetation (cascade effect). By genetically documenting the decline in predator DNA and then the increase in herbivore DNA and changes in flora, it is understood that the impact of the original crime is amplified ecologically. This helps to argue that such wildlife crimes are not "isolated incidents" but entail collateral damage to the ecosystem. The ecological justice response must therefore consider comprehensive measures (not just reintroducing the predator, but restoring the habitat conditions affected by herbivore overpopulation).

With this new approach, we are also broadening the range of questions we can ask in criminology. In this context, a criminologist might traditionally ask: ‘What laws have been broken and who is directly responsible?’ From a scientific-ecological perspective, we can also ask: ‘What ecological function has been damaged and what are the implications for the local community and the region?’ or ‘How do small everyday acts of pollution accumulate to have a quantifiable genetic impact in the long term?’ ( Agnew, 2020). This last point is of great importance. Agnew (2020) highlights how everyday acts of modern life (driving cars, consuming plastics, using pesticides in the garden) add up to a global phenomenon of gradual ecocide. A conceptual analysis enriched by science allows us to glimpse systemic solutions: not just punishing the offender, but changing the cultural, industrial and regulatory patterns that create the context for the damage.

Furthermore, this synergy between science and criminology opens the door to the democratisation of environmental information. To the extent that genetic data is objective and can be presented in an understandable way (biodiversity heat maps, DNA-based biotic integrity indices, etc.), affected communities and environmental movements can use it as a tool for reporting and participation. This fits in with applied green criminology, which seeks to "give voice" to invisible victims (including nature, but also silenced human communities). For example, citizen groups can carry out or demand eDNA monitoring in their local rivers to officially prove what they empirically suspected—that a certain industry is degrading aquatic life—and thus press for justice. Citizen science initiatives already exist where volunteers collect environmental DNA samples to map urban biodiversity, for example, empowering civil society with direct knowledge of their environment ( Bunce & Freeth, 2022). The confluence of this informed activism and green criminology produces a breeding ground for social change: when local people have tangible evidence of the deterioration (or improvement) of their ecosystem, they can negotiate remediation or conservation measures more forcefully with the authorities. In this sense, the approach discussed here is not merely theoretical but has practical potential: it brings science closer to the realm of community ecological justice.

### Towards comprehensive ecological justice

The integration of molecular genetics into green criminology not only helps us diagnose environmental harm but also strengthens the mechanisms for effective ecological justice. In this sense, we can outline several ways in which this comprehensive approach contributes to justice. For example, data on which biotic components have been affected could guide restorative actions. If analyses reveal the disappearance of certain fish and molluscs after a spill, remediation should include restocking of those species once the habitat has been decontaminated. In addition, post-restoration genetic monitoring serves to measure the success of the measures ( Bunce & Freeth, 2022), ensuring that justice is not just a statement but is verified in the actual recovery of the ecosystem. This is analogous to monitoring the rehabilitation of a human victim after a crime, except that here the "victim" is collective/ecological and its healing is measured in ecological parameters.

The availability of clear genetic information about the state of an environment allows citizens and local communities to be included in the justice process. For example, community boards can understand a report that says "the DNA of our river shows 30% less diversity than five years ago, and traces of salamanders have completely disappeared," and based on that, demand action from the authorities. Ecological justice has a social empowerment component, and science provides tools for this. At the same time, in restorative processes, involving the community in tasks such as sample collection or environmental monitoring increases the legitimacy and educational effect of justice (collective ecological awareness is generated).

As mentioned above, the prospect of detection reduces the temptation to commit environmental offences. But beyond classic deterrence (fear of punishment), there is an element of moral responsibility here. When companies or individuals know exactly what the impact of their actions is (because they are presented with scientific evidence), it is more difficult for them to justify themselves on the grounds of ignorance. Green criminology seeks precisely this cultural change: for actors to internalise the damage they cause. Science makes this explicit and therefore facilitates accountability processes.

At a more macro level, the integration of these fields informs the formulation of more effective public policies. A state that incorporates genetic monitoring into its environmental management will be able to identify previously ignored sources of deterioration and prioritise preventive actions in critical areas. This is in line with the principle of ecological precaution: acting in advance when there are signs of damage. Zero net biodiversity loss policies, for example, can rely on iDNA indicators to evaluate development projects and require compensation if losses are detected. To the extent that green criminology influences the political agenda (e.g., by recommending the criminalisation of certain behaviours or improved enforcement), having up-to-date data will make such recommendations more persuasive.

Many environmental problems transcend borders. The standardisation of genetic methods allows for global comparability. If researchers from different countries report similar genetic reductions in coral ecosystems, this reinforces the call for coordinated international action against, say, climate change or ocean acidification. Here, green criminology draws on scientific diplomacy: data creates a common language for understanding global damage. Ecological justice at the international level is driven by this kind of scientific consensus, as was the case with the ozone hole (where incontrovertible chemical evidence led to the Montreal Protocol). We can imagine that genomic evidence of the sixth mass extinction of species will contribute to a consensus for Paris Protocol-type agreements focused on biodiversity, establishing shared obligations for ecological restoration.

The synergy between green criminology, molecular genetics and ecological justice is moving us towards a paradigm of comprehensive ecological justice, where prevention, restoration and participation go hand in hand, informed by sound scientific knowledge and guided by values of respect for the Earth. This paradigm recognises the interdependence between human well-being and ecosystem health, with a view to ensuring a single planetary health, breaking down the false dichotomies that have historically separated 'the environmental' from 'the social' or 'the criminal' from 'the ecological' ( Alonso & South, 2025). Of course, the transition to this paradigm is not without its difficulties. It requires political will, investment in science, and training for justice operators on environmental issues, as well as efforts to overcome the inertia characteristic of narrow legal frameworks. However, the benefits are clear given the magnitude of the current environmental challenge. A fragmented approach – whether purely punitive or merely scientific without an ethical framework – would be insufficient. The convergence analysed here offers a promising route for articulating knowledge and action.

## Conclusions

This contribution has refocused the debate on environmental harm towards a new theoretical perspective with ecocentric positions. We have done this by integrating green criminology, molecular genetics and ecological justice. This new vision at different levels has shaped a multidisciplinary and holistic prism for understanding ecological damage not as a diffuse or complex problem, but as a concrete, measurable and morally relevant reality that challenges traditional paradigms of law and criminology.

Firstly, this perspective has provided us with an approach that broadens the limits of what we consider to be crime and victimisation. Ecosystems and non-human beings are now considered subjects worthy of legal protection, and attacks or aggression against them, whether illegal or legal, constitute crimes that must be prevented and punished according to the damage caused. The critique of legal anthropocentrism and the incorporation of ecological harm provide us with a new tool, in this case a new language that allows us to name and denounce injustices that previously went unnoticed or were minimised. Green criminology, backed by critical theories, makes it clear that many of the worst ‘crimes’ against the Earth do not come from isolated individuals, but from unsustainable economic and political structures; recognising this directs solution strategies towards systemic changes and not just individual cases.

Secondly, techniques used in molecular genetics provide a new tool to support research in green criminology by providing empirical evidence of quantified environmental harm. In the face of certain activities that may pose a risk to ecosystems, these techniques allow us, among other things, to identify whether genes are disappearing, whether there is a damaged genome or whether there are traces of disappearing biodiversity. In other words, science provides material evidence of ecological crimes. We have seen how techniques such as eDNA can detect and quantify ecological transformations induced by pollutants, from species loss to profound alterations in biotic communities, making visible what was previously hidden. This synergy improves diagnosis (early identification of damage), prognosis (anticipation of cascading effects, ecological points of no return) and prescription (design of corrective measures based on ecology). Molecular genetics once again becomes an ally of justice, but in this case of ecological justice.

Thirdly, the principle of ecological justice has been reaffirmed as the ethical compass that should guide and orient the response to damage. This principle urges us to place the restoration of ecological balance and the protection of the integrity of life systems as a fundamental objective. Consequently, the repair of environmental harm ceases to be a secondary issue and takes centre stage. The inclusion of ecological justice also implies adopting a long-term perspective and intergenerational equity: present decisions must be evaluated in light of their impacts on future generations, both human and non-human. The analysis developed emphasises that the pursuit of ecological justice is not at odds with social justice, but rather that the two complement each other: there can be no lasting human well-being on a degraded planet, nor effective protection of nature that ignores human needs. Solutions must therefore seek to simultaneously restore ecosystems and improve the quality of life of the communities that depend on them, an objective consistent with the emerging global philosophy of ‘One Health’ and climate justice. All of the above raises new fronts to be addressed with important practical implications, as the various institutions with responsibility for the environment must adapt to this new perspective.

This involves incorporating new resources to develop research that integrates these types of methods and tools. Similarly, it is urgent to update regulatory frameworks to include rules adapted to this reality and damage assessment criteria based on ecological indicators. Only then can genetic and ecological information be translated into sound legal decisions, such as ordering the suspension of activities before irreparable damage occurs or imposing comprehensive environmental restoration obligations in convictions. Some legal systems are already taking steps in this direction, recognising the rights of nature (e.g. Ecuador and Bolivia) or integrating the concept of pure environmental harm into their civil and criminal legislation; this trend should be deepened and universalised, based on available scientific knowledge.

In addition to all of the above, this analysis also highlights the importance of education and communication in order to increase the effective protection of nature. For this reason, the dissemination of information is of great importance, especially when incorporating these techniques; we must include advances in genetics and their importance among justice operators, legislators and the general public, which is essential for creating an informed culture of ecological respect. When a broad spectrum of society understands, for example, what the DNA sequencing of our rivers or forests tells us, it will be easier to reach a consensus on ambitious environmental public policies. Green criminology can contribute to this task by translating scientific findings into understandable narratives that raise awareness of the value of ecosystems and the risks of their loss, as this work has been ongoing for the last few decades (see the work of Morelle-Hungría & Serra-Palao, 2024; Morelle-Hungría & Serra-Palao, 2025).

## Ethics and consents

Ethical approval and consent were not required.

## Data Availability

No data associated with this article.
