# Molecular genetics as evidence of environmental harm in ecocriminological analysis

**DOI:** 10.12688/openreseurope.21126.2

**Published:** 2025-10-27

**Authors:** Esteban Morelle-Hungría

**Affiliations:** 1 Senior Lecturer in Criminology. Public Law Department, University Jaume I, Castellón de la Plana, Valencian Community, Spain

**Keywords:** eDNA, environmental harm, molecular genetics, ecological damage, green criminology

## Abstract

This research focuses on the potential of molecular genetics as a tool that can complement the assessment and evaluation of environmental damage from the perspective of green criminology or ecocriminology. This would have an impact on the effectiveness and efficiency of the mechanisms established for assessing the damage caused to ecosystems. We are facing a planetary crisis with the risk of ecosystem collapse, so it is proposed to overcome the limitations that we can identify in traditional criminal law by adopting an ecocentric approach reinforced with innovative mechanisms provided by science. This requires, among other things, recognising the intrinsic value of nature and committing to ecological justice. Molecular genetics methods, such as environmental DNA, metagenomics and population genetics, allow us to visualise the biological and ecological transformations induced by pollutants, even when these are invisible to the naked eye. These techniques provide objective and quantifiable data on biodiversity loss, changes in community composition and even possible genotoxic effects. Therefore, these molecular tests can complement preventive and restorative measures in environmental crimes. By fostering dialogue between science, law, and ethics, this study advocates for an integrated paradigm of environmental damage analysis in which molecular genetics enhances our ability to detect, understand, and legally address ecological damage. The convergence of green criminology, molecular genetics, and ecological justice reorients institutional responses toward restoring ecosystem integrity and defending the rights of nature.

## Introduction

We are witnessing a planetary crisis in which the current risks and problems facing all species are unprecedented ( Marteache Solans, 2024). This situation stems, among other things, from human activity linked to a model mired in excessive consumerism, which implicitly involves the accelerated loss of natural resources. The planet in general, but particularly biodiversity and ecosystems, are the most affected, and therefore planetary boundaries represent a new perspective for shifting towards a comprehensive and holistic model of integral protection of the planetary balance, given that “one health” is being affected ( Alonso & South, 2025; Morelle-Hungría, 2022). The scientific literature in this area paints a devastating picture. For example, around one million species may be at risk of extinction in the coming decades, a milestone in history as this number has never before been quantified ( IPBES, 2019). To this must be added the consequences and impacts derived from global problems such as global warming ( IPCC, 2021). This highlights the level of anthropogenic pressure exerted on the planet's ecological systems ( IPCC, 2021). In this context, traditional criminology – focused on conventional crimes and an anthropocentric approach – is ineffective in understanding and addressing the complexity of ecological damage ( South, 2014). Green criminology is a perspective of great interest because, among other things, it allows criminology to focus on environmental harm as an object of study. For example, reaching extremes never seen before many of the actions that are harmful or risky to nature are classified as lawful activities or even carried out by the administration itself ( Lynch & Stretesky, 2014; Morelle-Hungría, 2020).

This criminological perspective implies essential transformations are needed for the comprehensive analysis of ecological damage. On the one hand, it broadens and redesigns the notion of crime by emphasising the concept of harm. Beyond the formal violation of criminal laws, it is argued that acts or omissions that cause negative damage to ecosystems, fauna, flora and even ecological processes should be considered crimes ( Brisman, 2014; Lynch & Stretesky, 2014). Similarly, it broadens the concept of victim to include non-human species, the environment and even the entire planet as subjects worthy of protection ( Brisman, 2014; Morelle-Hungría, 2023). Consequently, the category of environmental offender is no longer limited to the tangible individual who dumps waste or poaches but also encompasses structural actors and factors—such as corporations, states and even global economic dynamics—ultimately responsible for ecological collapse ( Brisman, 2014). Thanks to the green perspective, criminology provides us with a critical and holistic view that allows us to question the political, economic, and cultural roots of ecological degradation. Advances in molecular biology have opened up a new avenue for improving our comprehensive understanding of the damage caused. With the incorporation of tools and methods such as environmental DNA (eDNA), metagenomics, and population genetic analyses, it is possible to monitor the health of ecosystems with an unprecedented level of precision. These molecular techniques make it possible to identify the presence, absence, or even abundance of species within an ecosystem, and to detect early ecological changes caused by anthropogenic pressures. This opens up new lines of research for assessing ecosystem integrity and resilience ( Allen *et al.*, 2021; Bunce & Freeth, 2022; Dejean *et al.*, 2011; Goldberg *et al.*, 2016; Seymour *et al.*, 2021; Wineland *et al.*, 2019). This approach also enables the identification of a “molecular or biological footprint” that reveals the composition and dynamics of a given habitat without resorting to invasive forms of evidence collection ( Bunce & Freeth, 2022). Such techniques have been widely applied in ecology, conservation biology, and even forest management, providing a more holistic view of the biological networks that sustain ecosystems. For green criminology, molecular genetics offers a way to visualise ecological transformations—sometimes subtle, yet significant—caused by human activities, thus bridging the gap between scientific detection and legal recognition of environmental harm.

Another key conceptual axis in this analysis is ecological justice. Derived from an ecocentric worldview, ecological justice proposes that the moral community should extend beyond humanity to include non-human nature, recognising the intrinsic value of ecosystems and demanding their protection as a right in their own right ( White, 2018). We cannot ignore the fact that without ecosystems and biodiversity, life cannot be guaranteed, let alone human rights, including the right to a healthy environment, which is why this argument alone would be sufficient to grant fundamental and non-instrumental legal value ( Borrás-Pentinat, 2024). In order to achieve this recognition, most countries should implement the recognition of the rights of nature. In this regard, we agree with Borrás-Pentinat (2024, p. 235) when he states that this requires the intervention of both individual states and the European Union itself.

Unlike traditional environmental justice, which tends to focus on the equitable distribution of environmental harms among human groups, ecological justice focuses on the well-being of the natural environment itself and all the beings that comprise it, advocating for the repair of ecological damage itself and the restoration of lost balance ( Morelle-Hungría & Serra-Palao, 2024; Sollund, 2022). Within the framework of green criminology, ecological justice serves as a guiding principle that informs both our understanding of the problem (who or what is harmed when we degrade nature?) and our responses to it (how can we remedy that harm in a comprehensive way?). This approach rejects individualistic bioethical views—for example, debates focused exclusively on the rights of the human being involved—and transcends the traditional punitive paradigm, emphasising the restoration of ecological systems and the prevention of further harm as a measure of justice ( Di Ronco & South, 2025; Morelle-Hungría & Serra-Palao, 2024). This research develops a theoretical and conceptual analysis of ecological damage from the perspective of green criminology. We do so for the first time, integrating contributions from molecular genetics and intersecting with the notion of ecological justice. Next, we will focus on the theoretical framework, where we will first describe the foundations of green criminology and ecological justice. Subsequently, we will show how molecular genetics can contribute to broadening our understanding and analysis of environmental harm caused by human activities. In addition, we will explore the intersection between green criminology and molecular genetics in order to characterize ecological transformations, offering a systematic view of the damage caused by different activities.

## Theoretical framework

### Green criminology and environmental harm

The beginning of this criminological perspective, described as green, dates back to the early 1990s, in response to concerns about the serious environmental consequences of human conflicts, especially given that the approaches implemented were inadequate to deal with these situations ( Marteache Solans, 2024). It is recognized as the beginning of green criminology, proposing an ecologization of criminology as a new paradigm focused on human acts that cause damage to the environment, questioning the traditional indifference of criminology itself towards crimes without human victims ( Lynch, 1990) while South (1998: 226) suggested that “Green issues open up a wide range of possibilities for interdisciplinary work, both within the social sciences and with disciplines in the natural sciences”. This trend gained momentum in the following decades, consolidating itself theoretically through fundamental works (e.g. Brisman & South, 2013; Lynch & Stretesky, 2014; White & Heckenberg, 2014) that laid the foundations for a "non-anthropocentric" criminology ( South, 2014).

An essential starting point for green criminology is the critique of the anthropocentrism that dominates criminal law and traditional criminology ( García Ruiz & Morelle-Hungría, 2023). According to South (2014), the classic utilitarian view conceives of nature merely as a resource at the service of human interests, focusing on environmental harm only to the extent that it directly affects people or their property. This leaves a wide spectrum of systematic ecological aggression outside the criminological focus, especially where harm falls mainly on animals, forests, rivers or other natural elements without an immediate link to human victims ( South, 2014). Green criminology breaks with this limitation by asserting that the environment itself can be the victim of injustice and harm, and therefore the subject of criminological attention ( Brisman, 2014). This entails broadening the definition of "crime" beyond legal transgression to include any human action or omission that causes significant ecological harm, even if such action is legal under current regulations ( Vegh Weis, 2024). This approach allows for practices that also cause harm but are socially or legally tolerated – such as industrial discharges within permitted limits, "legal" deforestation, overfishing, carbon emissions that comply with lax regulations, etc. – but which cumulatively degrade the biosphere to such an extent that they may exceed activities considered illegal, including ( Colás Turégano & Morelle-Hungría, 2021; García Ruiz, 2022; García Ruiz & Morelle-Hungría, 2023; Morelle-Hungría, 2020). The central idea is that the parameter for considering something criminologically relevant is the harm caused, not just its legal status ( Lynch, 1990; Lynch & Stretesky, 2014).

Green criminology proposes analysing phenomena such as chronic pollution, biodiversity loss and climate change as forms of environmental crime or victimisation, even if they do not always fit into typical criminal categories. Lynch and Stretesky (2014) advocate a "green revolution" in criminology where "green harms" – a term that encompasses damage to ecosystems and animals – are taken as seriously as traditional crimes against people or property. This also implies questioning the power structures that allow such harms to occur. Many environmental crimes are not the work of isolated individuals but are linked to structural dynamics: economic development models, extractive policies, mass consumption and corporate cultures focused on immediate profit over sustainability ( South, 2014). Green criminology, influenced by critical criminology and the "treadmill of production" theory of environmental sociology, examines how global capitalism and the pursuit of perpetual economic growth contribute to the overexploitation of nature, creating systemic "environmental criminogens" ( Lynch & Stretesky, 2014). It identifies macro-structural factors as fundamental causes of ecological damage, such as polluting transnational corporations and international supply chains, among others ( South, 2017). This alone broadens the traditional focus on individual criminals to include white-collar crime and corporate crimes related to the environment ( Lynch *et al.*, 2020).

In this context, the green perspective is shaped by various theoretical frameworks. On the one hand, critical criminology and Marxist theories are integrated to understand the political and economic dimensions of the problem. On the other hand, it incorporates expanded victimology by considering a broader view of the victims of this type of crime in order to protect vulnerable communities and even nature itself ( Marteache Solans, 2024). In turn, connections with other disciplines further removed from criminology are taken into account: ecology, ethics, international law, and environmental justice studies. All of this shapes a multi-level, interdisciplinary approach aimed at comprehensively understanding environmental harm, its causes, and its consequences.

A relevant conceptual distinction within green criminology is that of "environmental justice" versus "ecological justice". Traditional environmental justice deals with the equitable distribution of environmental burdens among different human groups (e.g., ethnic minorities, poor communities disproportionately affected by pollution), while ecological justice—as we will see in detail below—extends the sphere of justice to nature itself. Both strands inform green criminology: on the one hand, there is concern about the unequal impacts of environmental degradation on vulnerable human populations (e.g., indigenous peoples affected by extractivism); on the other hand, it is recognised that the devastation of species and habitats poses a justice problem that transcends anthropocentrism ( White, 2013).

Green criminology provides an expanded theoretical and conceptual framework for analysing environmental harm: it defines such damage as a legitimate object of criminological study, questions legal boundaries and proposes an ecocentric view in which protecting ecological integrity becomes a central imperative ( Brisman, 2014). It is not just a matter of cataloguing new "environmental crimes", but of rethinking what we understand by crime, victim and justice in the Anthropocene era, where human actions threaten the biophysical stability of the planet ( South, 2014). Once this conceptual framework has been established from the perspective of green criminology, we are interested in how molecular genetics—including techniques such as environmental DNA (eDNA), metagenomics, and genetic analysis of populations—can complement and enhance this perspective, as it can provide us with solid scientific evidence of ecological change and environmental damage. These molecular approaches open up avenues that even allow us to detect patterns of biodiversity, changes in community composition, and even functional alterations in ecosystems as a result of anthropogenic pressures. Integrating this data into ecocriminal analysis allows us to strengthen the empirical basis for recognising and demonstrating ecological damage, helping to make the invisible dimensions of environmental damage visible from a scientific and legal perspective ( Allen *et al.*, 2021; Dejean *et al.*, 2011; Goldberg *et al.*, 2016; Seymour *et al.*, 2021; Wineland *et al.*, 2019).

### Environmental molecular genetics: making ecological damage visible

As we have already mentioned, molecular genetics applied to the environment allows us to integrate a set of techniques and approaches that use DNA, RNA or other genetic markers to study ecological components and processes. Environmental DNA (eDNA) analysis has become an effective technique for monitoring biodiversity and tracking ecological changes. eDNA is the genetic material that organisms leave behind or shed in their environment, such as skin cells, feces, hair, pollen, or spores. By collecting samples of water, soil, sediments or air and extracting this residual genetic material, it is possible to identify which species have been present in a given location, even without observing them directly ( Lewis *et al.*, 2024). This represents a paradigm shift in environmental monitoring: it is no longer essential to have sight of or capture specimens; the "genetic signature" they leave behind is enough to reveal their presence.

The proven uses of eDNA and other molecular tools in environmental science are numerous. These approaches have already been used for community-wide biodiversity assessments across most environmental matrices, including freshwater, marine systems, sediments, and even air. Single-species targeted assays are extremely useful and often more sensitive than traditional methods for the detection of rare, endangered, harmful, or invasive species ( Allen *et al.*, 2021; Dejean *et al.*, 2011; Goldberg *et al.*, 2016; Seymour *et al.*, 2021; Wineland *et al.*, 2019). They also allow monitoring at multiple temporal scales, enabling the detection of medium- and long-term changes in the species composition of ecosystems.

These types of techniques have solid scientific and academic backing. Authors such as Lewis *et al.* (2024) point out that the scientific community has gained confidence in molecular monitoring methods as their effectiveness has been confirmed in various contexts of conservation, wildlife management and climate change studies. Such is the degree of consolidation and growing confidence in this tool that its incorporation into forensic investigations of wildlife and other environmental crimes is now being explored, given its ability to provide robust biological evidence ( Lewis *et al.*, 2024). However, it is important to note that in this analysis we will not focus on the use of eDNA as evidence in legal proceedings, but rather on its value in understanding and highlighting ecological damage from a systemic perspective that can help us increase the effectiveness of protection mechanisms. As Fernández-Hernández (2024) points out, legal systems must be effective in ensuring that the legislative techniques used to incorporate new types of crimes related to nature are updated and refined.

How, then, does molecular genetics contribute to making environmental harm visible and characterising it? Firstly, by detecting biodiversity loss. Many environmental impacts manifest themselves not so much by the presence of something, but by the absence or decline of sensitive species. For example, certain aquatic insects may disappear from a river after it has been polluted with agrochemicals, or the diversity of soil microbes may collapse due to toxic spills. Traditionally, recognising these losses required lengthy field sampling and expert taxonomic knowledge. With these molecular techniques, it is sufficient to analyse water, soil, or air samples to determine whether genetic traces of certain species are disappearing or decreasing, thus allowing an early warning signal to be established ( Bunce & Freeth, 2022). In fact, eDNA offers much greater temporal and spatial resolution than traditional methods, allowing for near real-time monitoring of biota. This issue is interesting even for environmental criminal law itself or for green criminology, as many forms of environmental harm are gradual and cumulative; the ability to detect them at an early stage facilitates the assignment of responsibility and the adoption of preventive measures before major collapses occur.

Importantly, molecular approaches allow us to distinguish between biodiversity loss and the loss of bioindicator species—two related but distinct ecological processes. A decline in α-diversity (e.g., from 30 to 3 detected species) signals a severe biodiversity loss, while the disappearance of a key indicator species, even within a relatively stable community, can serve as an early warning of ecosystem stress or impending collapse ( Acharya-Patel *et al.*, 2025). Secondly, molecular genetics allows us to characterize changes in biological communities induced by external and internal disturbances. Not all pollutants kill life immediately; some generate ecological subtleties, such as changes in species composition (some proliferate, others decline). For example, chronic discharge of nutrients or heavy metals into an ecosystem may not exterminate all life, but it may favour opportunistic organisms (algae, tolerant bacteria) to the detriment of more sensitive species, profoundly altering the trophic web. These transformations can be detected throuhg eDNA metabarcoding and metagenomic analyses, which identify multiple species simultaneously and estimate their relative abundances. This makes it possible to profile “genetic signatures” of pollution—characteristic molecular patterns that reveal, for example, the dominant presence of microbes indicative of organic contamination, the absence of invertebrate bioindicators of clean water, or the anomalous abundance of metal-resistance genes in microbial communities. Such findings provide an objective description of the damage: they not only confirm that an impact occurred but also reveal how the local ecology has been transformed.

An illustrative case comes from the analysis of marine environments affected by authorised human activities. Morelle-Hungría and Serra-Palao (2024) studied the effect of brine discharged by desalination plants on a Mediterranean seagrass bed, *Posidonia oceanica,* a key species protected both at Community level and as a priority habitat. This study highlights the ecological damage caused by this legal discharge and emphasises the need to address it through ecological justice theory with mitigation and adaptation techniques to improve the effectiveness and efficiency of the facility ( Morelle-Hungría & Serra-Palao, 2024). In fact, the authors propose solutions based on environmental restorative justice, seeking to repair the affected ecosystem (e.g., mitigation of the discharge, restoration of the seagrass meadow) rather than simply sanctioning the plant operators ( Morelle-Hungría & Serra-Palao, 2024). With the implementation of eDNA and other molecular techniques in this context, it would be possible to further analyse the ecological impacts caused by these physicochemical changes in the affected ecosystems, as well as to establish timelines showing the cumulative effects of the impact. Additionally, it would allow for the identification of species most affected by such discharges, thereby enhancing the precision and value of environmental assessments.

Another contribution of molecular genetics is the detection of genotoxic effects in organisms exposed to pollutants. Genetic biomarkers indicate DNA damage in individuals or communities (e.g., high mutation rates, chromosomal aberrations, or changes in gene expression associated with chemical stress) ( Bickham *et al.*, 2000). Although this approach focuses more on individuals than on communities, its ecological implications are clear: a fish population slowly poisoned by mercury may not exhibit sudden mortality, but it will show DNA damage and reduced reproductive success, foreshadowing a population collapse. Environmental forensic genetics can therefore reveal this "invisible damage" by measuring, for example, the frequency of micronuclei in blood cells of wild animals from contaminated areas (an indicator of sublethal genetic damage). A promising area is the integration of genomic data to understand long-term systemic A promising and emerging area is the study of environmental RNA (eRNA) and metatranscriptomics, which allow researchers to assess ecosystem functioning and stress responses in real time. These methods can reveal which genes are actively expressed under pollutant exposure, providing insights into the functional state of ecosystems beyond species presence or absence. Molecular genetics techniques thus make it possible to assess and quantify ecological damage, providing a valuable complement to traditional assessments with tangible, measurable evidence.

They also enhance prevention mechanisms, as they enable the detection of pollutants or their effects before irreversible harm occurs, thereby improving proactive environmental protection. Recent studies have identified, for example, the presence of human DNA in water samples—highlighting the potential of these technologies to trace anthropogenic influences and link molecular evidence to human activities ( Goray *et al.*, 2024). From a green criminology perspective, these molecular tools could also serve as a deterrent within general prevention mechanisms, since offenders would know that the damage they cause leaves a genetic trace that can be detected and attributed, reducing their sense of impunity. As Bunce and Freeth (2022) note, eDNA and related molecular approaches have the potential to increase the visibility and understanding of the ecological consequences of our actions, thereby reinforcing ecological justice and the protection of nature’s rights.

### Ecological and restorative justice as guiding principles

Within the normative and ethical framework of green criminology, the concept of ecological justice occupies a central place. As mentioned above, ecological justice is based on the idea that nature has intrinsic value and that human beings have an obligation to respect ecological limits and the rights of other species and ecosystems to exist and thrive. This concept complements the notion of environmental justice—which typically focuses on unjust relationships between human groups and the environment—and expands it towards a biocentric or ecocentric ethic ( White, 2018).

In practical terms, embracing ecological justice implies three things for the analysis of environmental harm: recognition, responsibility and reparation.

Recognition means admitting that damage to the natural environment is in itself a wrong that must be recognised by our justice institutions, regardless of whether it can be translated into terms of human injury. For example, the disappearance of a coral reef or the extinction of a species has moral and legal relevance beyond any associated economic loss or impact on human well-being. In green criminology, this recognition is reflected in the proposal of concepts such as ecocide, understood as human acts that cause massive damage to the environment and deserve international criminal punishment in the same way as crimes against humanity ( Agnew, 2020; Higgins *et al.*, 2013). Although the debate on ecocide belongs to the field of emerging criminal law and we will not delve into its legal admissibility here, the mere discussion of it reflects a change in sensitivity: it is beginning to be accepted that devastating entire ecosystems is a fundamental injustice that must be prevented ( García Ruiz *et al.*, 2022).

Responsibility in terms of ecological justice means broadening the range of those obliged to answer for environmental harm. It is not limited to the individual who directly polluted a river but includes collective and generational responsibility. This links to the idea of intergenerational justice (leaving a healthy environment for future generations) and the responsibility of corporations and states ( Ruggiero & South, 2010). For example, industrialised countries and large polluting companies have a disproportionate responsibility for the global ecological crisis, and ecological justice – or an international law of ecocide - would demand reparations or compensation from them to the countries and communities most affected by environmental degradation ( Higgins *et al.*, 2013; Lynch *et al.*, 2013). It has been estimated that 75% of environmental harm to nature is caused by legal entities ( Cuerda Arnau, 2023). From this perspective, environmental impunity stems not only from a failure to enforce laws, but also from a world order that allows certain actors to profit while transferring environmental costs to third parties (often poor populations or nature itself). Green criminology analyses these power imbalances and attempts to highlight them as structural injustices.

Reparation implies that, in the face of proven environmental harm, the priority response of justice must be to restore, as far as possible, the ecological balance that has been disrupted. This connects with the philosophy of green restorative justice. Rather than focusing exclusively on punishing the offender, restorative justice seeks to involve all affected parties (including the community and even, metaphorically, the environmental victim represented by scientists or environmental advocates) in agreeing on actions to repair the damage and prevent its recurrence. For example, if a company dumped waste into a lake resulting in the elimination of species, an ecological-restorative justice approach would propose: 1) requiring the company to finance the cleanup and recovery of the lake (replanting species, reintroducing fauna, decontaminating sediments); 2) ensuring the participation of the local community in the supervision of such restoration; and 3) encouraging a change in the company's practices so that the event does not happen again, perhaps through technological changes or permanent monitoring. In many current legal systems, restorative measures are secondary to criminal or administrative sanctions; green criminology advocates reversing this priority, placing the healing of the ecosystem at the centre of the response to ecological crime ( García Ruiz & Morelle-Hungría, 2023; Varona, 2022).

The concept of ecological justice, therefore, reorients the very purpose of social reaction to environmental harm. It is no longer just a matter of punishing the violation of a rule (which, in traditional environmental crimes, often boils down to fines that companies consider "costs of doing business"), but of restoring ecological integrity and the harmonious relationship between humans and nature ( García Ruiz & Morelle-Hungria, 2023). This also ties in with acknowledgment of and need to preserve the knowledge of Indigenous peoples and Indigenous worldviews that conceive of human beings as part of a greater web of life to which respect and obligations are owed ( Marteache Solans, 2024). In contemporary green criminology, especially from global Southern perspectives, this notion is captured through the idea of combating "ecological discrimination"—the marginalisation of nature in our considerations of justice ( Goyes *et al.*, 2021; Goyes, 2023) —and promoting rights of nature in constitutions and laws.

By incorporating ecological justice as a guiding principle, our analysis of environmental harm is enriched in several ways. First, it offers an evaluative criterion for judging the seriousness of harm: for example, the disappearance of an entire species or the destruction of a unique ecosystem should be considered a major wrong, regardless of its economic value, because it means the irretrievable loss of an irreplaceable component of the Earth (an attack on "ecological integrity," this approach would say). Second, it clarifies the ultimate objective of criminal intervention: the goal is to safeguard and, when necessary, restore the balance of life systems, rather than satisfy an abstract desire for retribution. This does not exclude sanctions but makes them subordinate to the restorative end. Third, ecological justice strengthens the argument in favour of policies to prevent environmental harm: if we recognise the inherent rights or interests of nature, the state and society would have a proactive obligation to prevent their impairment (precautionary principle), not just to react once the damage has been done.

In short, ecological justice provides the ethical and legal compass for green criminology, and it is argued here that it can be fruitfully complemented by the technical and evidentiary tools of molecular genetics, fostering a more integrated understanding of harm. While molecular genetics enables the detection and quantification of ecological transformations—revealing biodiversity loss, community disruption, and genetic stress—ecological justice gives these findings moral and legal meaning by framing them within duties of recognition, responsibility, and restoration. Together, they bridge the gap between scientific evidence and normative response, strengthening the foundation for preventive and restorative approaches to environmental harm. In the next section, we analyse how the convergence of these dimensions—green criminology, molecular genetics, and ecological justice—enables a comprehensive model for understanding, evidencing, and addressing ecological damage.

## Discussion: Integrating science and criminology to highlight environmental harm

The combination of green criminology and molecular genetics, under the umbrella of ecological justice, offers us an innovative and powerful framework to understanding environmental harm in an ecological and systemic way. In this discussion, we address three key dimensions of this integration: (1) making environmental harm visible – how molecular evidence can bring to light previously hidden or disputed damage; (2) comprehensive and systemic understanding – how the joint criminological and molecular analysis reveals the interconnected complexity of environmental issues, from genetic to social levels; and (3) implications for ecological justice – how this integrated approach can strengthen the application of restorative and preventive principles within environmental law and policy.

### Making the invisible visible: molecular evidence of ecological damage

One of the historical obstacles to the legal protection of the environment, both at the criminal and administrative levels, has been the 'invisibility' or diffuse nature of much damage. Starting with the most obvious, the criminal sphere, where unlike a common crime (e.g., theft or assault) the consequence is immediate and tangible, ecological crimes often operate silently, gradually, and in a dispersed manner. For example, how can one prove that a decline in fish populations in a river is due to discharges from a factory located upstream? Or how can one demonstrate that a permitted practice, such as agriculture using agrochemicals, is causing the loss of pollinators in a region?

Thanks to molecular genetic techniques, it is now possible to establish stronger scientific correlations between polluting activities and ecological changes. If eDNA analysis in an ecosystem adjacent to an industrial area shows that sequences corresponding to certain sensitive species (amphibians, insects) have disappeared, coinciding with the presence of toxic compounds in the water, this genetic-environmental correlation can reinforce evidence of anthropogenic damage ( Lewis *et al.*, 2024). Similarly, genetic profiles can reveal specific signatures of pollution: for example, the predominant presence of bacterial hydrocarbon degradation genes in marine sediment suggests oil pollution, or the detection of harmful algae DNA in a water column may indicate eutrophication due to excess nutrients. These molecular markers serve as bioindicators, offering quantitative and objective data that are difficult to dismiss. Moreover, advances in environmental RNA (eRNA) and transcriptomic analyses now enable the identification of functional responses of communities to stressors, such as gene expression changes linked to exposure to pollutants or oxygen depletion.

### Systemic and interdisciplinary understanding of environmental harm

The integration of molecular genetics tools into the criminological analysis of environmental harm not only provides scientifically sound empirical data but also enriches our qualitative and quantitative understanding of damage. By adopting this approach, we can perceive the problem at multiple scales —from the microscopic and genetic to the macroecological and social— and understand their interrelationships.This is consistent with a truly systemic view, aligned with the complexity of today’s environmental challenges.

A notable aspect of this integration is the convergence of languages and concepts between the natural and social sciences in the study of environmental harm. For example, in ecology, we talk about the resilience of ecosystems to refer to their capacity to absorb disturbances and recover. In green criminology, this concept can be adapted to assess how resilient an environment is to certain human activities: molecular genetics can indicate, through measures of genetic variability, population structure, or functional redundancy (several species fulfilling similar roles), how vulnerable or resilient an ecosystem is to impact. A forest with high genetic diversity, for instance, is more likely to tolerate moderate logging than a genetically impoverished one. If genomic analyses reveal that the genetic variability of key populations has been severely eroded (e.g., mangrove trees that are virtually clones due to historical deforestation), this warns that the ecosystem has lost its adaptive capacity and that the next disturbance could be catastrophic. Green criminology, informed by such molecular data, can thus prioritise urgent protective measures. In this way, a biological indicator becomes a criminological and policy-relevant priority.

Similarly, the notions of trophic cascades or cascading effects from ecology can illuminate criminological assessments of environmental harm. The disappearance of a predator species through illegal poaching can trigger an uncontrolled proliferation of herbivores, which in turn decimate vegetation—a cascade effect. By documenting these changes through molecular evidence (for example, a decline in predator DNA, a rise in herbivore DNA, and shifts in plant genetic diversity), the amplification of harm becomes visible. This molecular record strengthens the argument that such wildlife crimes are not “isolated incidents” but produce extensive collateral damage to ecosystems. The ecological justice response must therefore adopt comprehensive restoration measures—not only reintroducing the predator but also rehabilitating the habitat altered by herbivore overpopulation.

With this new approach, we also expand the scope of criminological inquiry. In this context, a criminologist might traditionally ask: ‘What laws have been broken and who is directly responsible?’ From a scientific-ecological perspective, we can add: ‘What ecological function has been damaged and what are the implications for the local community and the region?’ or ‘How do small everyday acts of pollution accumulate to have a quantifiable genetic impact in the long term?’ ( Agnew, 2020). This last point is of great importance. Agnew (2020) highlights how everyday acts of modern life (driving cars, consuming plastics, using pesticides in the garden) add up to a global phenomenon of gradual ecocide. A conceptual analysis enriched by science allows us to glimpse systemic solutions: not just punishing the offender, but changing the cultural, industrial and regulatory patterns that create the context for the damage.

Furthermore, this synergy between science and criminology opens the door to the democratisation of environmental information. Because molecular and genetic data can be presented in accessible formats—such as biodiversity heat maps or DNA-based biotic integrity indices—affected communities and environmental movements can use them as tools for advocacy and participation. This aligns with applied green criminology, which seeks to "give voice" to invisible victims, both human and non-human species. For example, citizen groups can carry out or demand molecular monitoring in their local rivers to officially prove what they empirically suspected—that a certain industry is degrading aquatic life—and thus press for justice. Citizen science initiatives already exist in which volunteers collect environmental DNA samples to map urban biodiversity, empowering civil society with direct and tangible ecological knowledge ( Bunce & Freeth, 2022). The confluence of this informed activism and green criminology produces a breeding ground for social change: when local people have tangible evidence of the deterioration (or improvement) of their ecosystem, they can negotiate remediation or conservation measures more forcefully with the authorities. In this sense, the approach discussed here is not merely theoretical but has strong practical potential, bridging science, justice, and community action in pursuit of ecological integrity.

### Towards comprehensive ecological justice

The integration of molecular genetics into green criminology not only helps us diagnose environmental harm but also strengthens the mechanisms for effective ecological justice. In this sense, we can outline several ways in which this comprehensive approach contributes to justice. For example, data on which biotic components have been affected could guide restorative actions. If analyses reveal the disappearance of certain fish and molluscs after a spill, remediation should include restocking of those species once the habitat has been decontaminated. In addition, post-restoration molecular or genetic monitoring serves to measure the success of the measures ( Bunce & Freeth, 2022), ensuring that justice is not just a statement but is verified in the actual recovery of the ecosystem. This is analogous to monitoring the rehabilitation of a human victim after a crime, except that here the "victim" is collective/ecological and its healing is measured in ecological parameters.

The availability of clear molecular information about the state of an environment allows citizens and local communities to be included in the justice process. For example, community boards can understand a report that says "the genetic data of our river shows 30% less diversity than five years ago, and traces of salamanders have completely disappeared," and based on that, demand action from the authorities. Ecological justice has a social empowerment component, and science provides tools for this. At the same time, in restorative processes, involving the community in tasks such as sample collection or environmental monitoring increases the legitimacy and educational effect of justice (collective ecological awareness is fostered).

As mentioned above, the prospect of detection reduces the temptation to commit environmental offences. But beyond classic deterrence (fear of punishment), there is an element of moral responsibility here. When companies or individuals know exactly what the impact of their actions is (because they are presented with scientific and genetic evidence), it is more difficult for them to justify themselves on the grounds of ignorance. Green criminology seeks precisely this cultural change: for actors to internalise the damage they cause. Science makes this explicit and therefore facilitates accountability processes.

At a more macro level, the integration of these fields informs the formulation of more effective public policies. A state that incorporates genetic or molecular monitoring into its environmental management will be able to identify previously ignored sources of deterioration and prioritise preventive actions in critical areas. This is in line with the principle of ecological precaution: acting in advance when there are signs of damage. Zero net biodiversity loss policies, for example, can rely on molecular indicators to evaluate development projects and require compensation if losses are detected. To the extent that green criminology influences the political agenda (e.g., by recommending the criminalisation of certain behaviours or improved enforcement), having up-to-date molecular and ecological data will make such recommendations more persuasive.

Many environmental problems transcend borders. The standardisation of molecular and genetic methods allows for global comparability. If researchers from different countries report similar genetic reductions in coral ecosystems, this reinforces the call for coordinated international action against, say, climate change or ocean acidification. Here, green criminology draws on scientific diplomacy: data creates a common language for understanding global damage. Ecological justice at the international level is driven by this kind of scientific consensus, as was the case with the ozone hole (where incontrovertible chemical evidence led to the Montreal Protocol). We can imagine that genomic evidence of the sixth mass extinction of species will contribute to a consensus for Paris Protocol-type agreements focused on biodiversity, establishing shared obligations for ecological restoration.

The synergy between green criminology, molecular genetics and ecological justice is moving us towards a paradigm of comprehensive ecological justice, where prevention, restoration and participation go hand in hand, informed by sound scientific knowledge and guided by values of respect for the Earth. This paradigm recognises the interdependence between human well-being and ecosystem health, with a view to ensuring a planetary ecological health, breaking down the false dichotomies that have historically separated 'the environmental' from 'the social' or 'the criminal' from 'the ecological' ( Alonso & South, 2025). Of course, the transition to this paradigm is not without its difficulties. It requires political will, investment in science, and training for justice operators on environmental issues, as well as efforts to overcome the inertia characteristic of narrow legal frameworks. However, the benefits are clear given the magnitude of the current environmental challenge. A fragmented approach – whether purely punitive or merely scientific without an ethical framework – would be insufficient. The convergence analysed here offers a promising route for articulating knowledge and action.

## Conclusions

This contribution has refocused the debate on environmental harm towards a new theoretical perspective with ecocentric positions. We have done this by integrating green criminology, molecular genetics and ecological justice. This new vision at different levels has shaped a multidisciplinary and holistic prism for understanding ecological damage not as a diffuse or complex problem, but as a concrete, measurable and morally relevant reality that challenges traditional paradigms of law and criminology.

Firstly, this perspective has provided us with an approach that broadens the limits of what we consider to be crime and victimisation. Ecosystems and non-human beings are now considered subjects worthy of legal protection, and attacks or aggression against them, whether illegal or legal, constitute crimes that must be prevented and punished according to the damage caused. The critique of legal anthropocentrism and the incorporation of ecological harm provide us with a new tool, in this case a new language that allows us to name and denounce injustices that previously went unnoticed or were minimised. Green criminology, backed by critical theories, makes it clear that many of the worst ‘crimes’ against the Earth do not come from isolated individuals, but from unsustainable economic and political structures; recognising this directs solution strategies towards systemic changes and not just individual cases.

Secondly, the techniques and methods of molecular genetics provide a new tool to support research in green criminology by providing empirical evidence of quantified environmental harm. In the face of certain activities that may pose a risk to ecosystems, these techniques allow us, among other things, to identify species or genes are disappearing, whether genomic integrity has been compromised, or whether molecular traces reveal a decline in biodiversity. In other words, science provides material evidence of ecological crimes. We have seen how molecular approaches such as eDNA, metagenomics, or population genetics, can detect and quantify ecological transformations induced by pollutants, from species loss to profound alterations in biotic communities, making visible what was previously hidden. This synergy improves diagnosis (early identification of damage), prognosis (anticipation of cascading effects, ecological points of no return) and prescription (design of corrective measures based on ecology). Molecular genetics once again becomes an ally of justice, particularly of ecological justice.

Thirdly, the principle of ecological justice has been reaffirmed as the ethical compass that should guide and orient the response to damage. This principle urges us to place the restoration of ecological balance and the protection of the integrity of life systems as a fundamental objective. Consequently, the repair of environmental harm ceases to be a secondary issue and takes centre stage. The inclusion of ecological justice also implies adopting a long-term perspective and intergenerational equity: present decisions must be evaluated in light of their impacts on future generations, both human and non-human. The analysis developed emphasises that the pursuit of ecological justice is not at odds with social justice, but rather that the two complement each other: there can be no lasting human well-being on a degraded planet, nor effective protection of nature that ignores human needs. Solutions must therefore seek to simultaneously restore ecosystems and improve the quality of life of the communities that depend on them, an objective consistent with the emerging global philosophy of ‘One Health’ and climate justice. All of the above raises new fronts to be addressed with important practical implications, as the various institutions with responsibility for the environment must adapt to this new perspective.

This involves incorporating new resources to develop research that integrates these types of methods and tools. Similarly, it is urgent to update regulatory frameworks to include rules adapted to this reality and damage assessment criteria based on ecological indicators. Only then can genetic and ecological information be translated into sound legal decisions, such as ordering the suspension of activities before irreparable damage occurs or imposing comprehensive environmental restoration obligations in convictions. Some legal systems are already taking steps in this direction, recognising the rights of nature (e.g. Ecuador and Bolivia) or integrating the concept of pure environmental harm into their civil and criminal legislation; this trend should be deepened and universalised, based on available scientific knowledge.

In addition to all of the above, this analysis also highlights the importance of education and communication in order to increase the effective protection of nature. For this reason, the dissemination of information is of great importance, especially when incorporating these techniques; we must include advances in genetics and their importance among justice operators, legislators and the general public, which is essential for creating an informed culture of ecological respect. When a broad spectrum of society understands, for example, what the genetic sequencing or molecular monitoring of our rivers or forests tells us, it will be easier to reach a consensus on ambitious environmental public policies. Green criminology can contribute to this task by translating scientific findings into understandable narratives that raise awareness of the value of ecosystems and the risks of their loss, as this work has been ongoing for the last few decades (see the work of Morelle-Hungría & Serra-Palao, 2024; Morelle-Hungría & Serra-Palao, 2025).

## Ethics and consents

Ethical approval and consent were not required.

## Data Availability

No data associated with this article.
