# Supplementary material for: Molecular genetics as evidence of environmental harm in ecocriminological analysis
Source: Open Res Eur. 2025 Oct 27;5:244. Originally published 2025 Aug 18. [Version 2] doi: 10.12688/openreseurope.21126.2 (PMC12624261; doi:10.12688/openreseurope.21126.2)
Supplement: Peer review history

# Reviewer report: Reviewer response for version 2

**DOI:** 10.21956/openreseurope.23378.r62874

**Published:** 2025-11-17

**Authors:** Neha Acharya-Patel

**Affiliations:** 1 University of Victoria, British Columbia, Canada

I am happy with the changes the author has made in response to my previous comments.

If applicable, is the statistical analysis and its interpretation appropriate?

Not applicable

Is the study design appropriate and is the work technically sound?

Yes

Is the work clearly and accurately presented and does it engage with the current literature?

Yes

Are the conclusions drawn adequately supported by the results?

Partly

Are sufficient details of methods and analysis provided to allow replication by others?

Not applicable

Are all the source data and materials underlying the results available?

No source data required

Reviewer Expertise:

NA

I confirm that I have read this submission and believe that I have an appropriate level of expertise to confirm that it is of an acceptable scientific standard.

# Reviewer report: Reviewer response for version 2

**DOI:** 10.21956/openreseurope.23378.r62875

**Published:** 2025-11-12

**Authors:** Jose Manuel Morante-Redolat

**Affiliations:** 1 Universitat de València, Valencia, Spain

I have carefully reviewed the revised version of the manuscript, and I believe the author has addressed the main concerns raised in the previous round very effectively.

 The paper now reads with greater clarity and coherence, and the argument develops in a smoother and more structured way. Repetitive sections have been significantly reduced, and the theoretical discussion feels more focused.

 The author has also strengthened the presentation of the paper’s main contribution, the integration of molecular genetics with green criminology and ecological justice. which now stands out clearly from the beginning.

 The new title accurately reflects the broader scope of the analysis, and the inclusion of practical examples and updated references adds depth and credibility to the work.

 The discussion and conclusion sections are now more cohesive, linking the scientific dimension of molecular tools with their criminological and legal implications in a convincing way.

 Overall, this is a much-improved and mature version of the manuscript. I consider that it now meets the standards for publication.

If applicable, is the statistical analysis and its interpretation appropriate?

Not applicable

Is the study design appropriate and is the work technically sound?

Yes

Is the work clearly and accurately presented and does it engage with the current literature?

Partly

Are the conclusions drawn adequately supported by the results?

Partly

Are sufficient details of methods and analysis provided to allow replication by others?

Not applicable

Are all the source data and materials underlying the results available?

No source data required

Reviewer Expertise:

Molecular and Cellular Biology. Genetics. DNA analysis.

I confirm that I have read this submission and believe that I have an appropriate level of expertise to confirm that it is of an acceptable scientific standard.

# Reviewer report: Reviewer response for version 1

**DOI:** 10.21956/openreseurope.22852.r61544

**Published:** 2025-10-20

**Authors:** Jose Manuel Morante-Redolat

**Affiliations:** 1 Universitat de València, Valencia, Spain

**Summary of the Paper**

 The manuscript explores how molecular genetics, and in particular environmental DNA (eDNA) analysis, can be applied to the study of environmental harm within the framework of green criminology. It argues that combining scientific tools such as eDNA analysis with legal and criminological perspectives can make ecological damage more visible and quantifiable. The author suggests that this integration supports a shift toward an ecocentric view of justice that recognizes the intrinsic value of nature and promotes the restoration of ecosystems rather than only the punishment of offenders. The paper brings together three key concepts: green criminology, molecular genetics, and ecological justice, to propose a interdisciplinary model for assessing and responding to environmental harm.

**My General Assessment**

 This manuscript presents a genuinely interesting and original contribution to the field of green criminology. It explores how molecular genetics, especially the analysis of eDNA, can help us better understand and evaluate environmental harm. The idea of connecting molecular science with criminological and legal analysis is both innovative and timely. It’s also handled with care, supported by a strong theoretical foundation and relevant literature.

 The paper is clearly the product of deep reflection and interdisciplinary effort. It moves smoothly between scientific, ethical, and legal discussions, and the author writes with conviction and clarity.  Most of my comments aim to help make the argument flow more naturally and reduce repetition, so the originality of the work stands out even more.

**Major Comments**

1. **Repetition and Flow**
  Some sections revisit the same ideas several times, particularly around the critique of anthropocentrism, the value of nature, and the difference between environmental and ecological justice. These are important concepts, but the paper would benefit from condensing them a bit. Streamlining these parts, especially in the *Introduction*, *Theoretical Framework*, and *Ecological Justice* sections, would make the argument more direct and easier to follow.
2. **Clarifying the Novelty**
  The integration of molecular genetics with ecocriminological thinking is, in my opinion, the paper’s most original aspect. It might help to highlight this more clearly at the beginning and again at the end. In other words, tell the reader right away what is new about this approach, and remind them of that contribution in the conclusion. On the other hand, the actual definition of  eDNA is first introduced in the text too late (second section of the *Theoretical framework*) but it is used from the beginning. I think it would be useful to define it as soon as possible in the text. 
3. **Title and terminological clarification**
  I feel that, at times, the text uses *eDNA* to refer both to the genetic material itself and as a synonym for *eDNA analysis*, which might be confusing for the reader. This also occurs in the title. You could consider something along the lines of: *“The use of molecular genetics to assess environmental harm: integrating eDNA into ecocriminological analysis.”*
4. **Interdisciplinary Integration**
  The theoretical synthesis is excellent, but it could go one step further by showing how these ideas might work in practice. How, for instance, could molecular data be used in real legal cases or in restorative justice settings? What kind of policies or preventive measures could arise from this type of analysis? Adding a short example, even a hypothetical one, could help make that connection more tangible.
5. **Conclusion**
  The conclusion is well written and rich in content, but it could be slightly shorter and more focused. Summarizing the main insights more directly would make the final message stronger. I would suggest ending with a clear statement of what the paper contributes and why that matters for both science and law.

**Minor Comments**

- The English is very good overall, though some sentences are quite long. Shortening or splitting a few of them would make the text lighter and more engaging.
- I understand that the author's intention was to say that eDNA includes genetic material found in 'feces' and not in 'fences'.
- eDNA is defined at least twice across the text. It should be defined just the first time: Environmental DNA (eDNA).
- The paper alternates between “green criminology” and “ecocriminology.” It might be useful to clarify whether these terms are meant to be synonymous or if one has a more specific meaning.
- Breaking up some long paragraphs would help readability, especially in the theoretical sections.

If applicable, is the statistical analysis and its interpretation appropriate?

Not applicable

Is the study design appropriate and is the work technically sound?

Yes

Is the work clearly and accurately presented and does it engage with the current literature?

Partly

Are the conclusions drawn adequately supported by the results?

Partly

Are sufficient details of methods and analysis provided to allow replication by others?

Not applicable

Are all the source data and materials underlying the results available?

No source data required

Reviewer Expertise:

Molecular and Cellular Biology. Genetics. DNA analysis.

I confirm that I have read this submission and believe that I have an appropriate level of expertise to confirm that it is of an acceptable scientific standard, however I have significant reservations, as outlined above.

# Reviewer report: Reviewer response for version 1

**DOI:** 10.21956/openreseurope.22852.r60705

**Published:** 2025-10-07

**Authors:** Neha Acharya-Patel

**Affiliations:** 1 University of Victoria, British Columbia, Canada

Below I have provided a high level summary of the work, with some suggested improvements on the parts concerning my expertise. 

 Overall I thought this paper was extremely fascinating and exciting regarding the potential legal uses for molecular data. As someone unfamiliar with environmental law, I thought this paper did a wonderful job of explaining complex concepts through accessible language and clear examples. 

 I do think however, that maybe removing the focus from eDNA in the title may be good. Maybe change to: "The Use of molecular genetics as a method for assessing environmental harm and ecological damage in the ecocriminological analysis"

 The only reason I suggest this, is that only one of your paragraphs focuses specifically on eDNA. You speak about metagenomics, and population genetics, and other molecular methods that are highly relevant as well. 

 But whatever you choose will be good! I really enjoyed reading this!

 Summary:

 Theoretical Framework Section:

 This section expands upon the concept of Green Criminology and how its integration into legal frameworks would pivot the central view of criminology away from a purely anthropocentric viewpoint.

- The key parameter to whether something is criminologically relevant is the harm caused, not the legal status
- This legal viewpoint is more interdisciplinary and benefits from fields such as : ecology, ethics, international law, and environmental justice studies.
- Environmental Justice refers to equitable distribution of environmental harm amongst all human demographics, whereas Ecological Justice refers solely to environmental harm independent of human harm

Environmental molecular genetic: making ecological damage visible

- “eDNA is the genetic material that organisms leave behind or shed in their environment, such as skin cells, fences, hair, pollen, spores, etc. “
  - I am confused what is meant by fences? Maybe consider removing that?
- “The proven uses of eDNA in environmental science are numerous. There are already promising results, for example, for conducting biodiversity inventories in certain ecosystems: rivers, lakes, and even oceans, detecting species that are difficult to sample using other methods. They can also be used to monitor protected or endangered species, for example, by searching for their DNA in the water to confirm whether or not they inhabit that area. In addition, it has proved very positive for the early detection of invasive alien species and pathogens before they proliferate uncontrollably. Furthermore, the use of this technique allows studies to be carried out at different time scales to monitor medium- and long-term changes in the species inhabiting a given ecosystem.”
  - I would improve this paragraph to be more accurate. Eg. eDNA has already been used for community wide biodiversity assessments in most environmental matrices, including freshwater, saltwater, sediments and even air. Using single species targeted approaches are extremely useful, and often more sensitive than traditional methods for the detection of rare, endangered, or harmful or invasive species.
  - Also add more citations eg. Seymour et al., 2021, Allen et al., 2021; Dejean et al., 2012; Goldberg et al., 2016; Wineland et al., 2019
- For Paragraphs four, five and six, I would clarify certain arguments better. You explain the concept of community compositional changes very well, however it also seems that you equate biodiversity loss with the loss of bioindicator species. I would better define the differences between those two. Eg. A loss of alpha biodiversity (eg. Going from 30 species detected to 3 species detected) is generally a bad sign. However, losing one key indicator species may not be as dramatic in metabarcoding studies, but could have a larger negative impact on the ecosystem, or may be a warning sign of further damage to come.
  - https://www.sciencedirect.com/science/article/pii/S0025326X25005119
- Paragraph 8 is very compelling, and provides nice examples. Might also be worthwhile to speak on the growing field of eRNA research, and metatranscriptomics as areas of research that will also be able to assess ecosystem functioning in response to stressors.
- Paragraph 9 : I am unclear what you are implying with this sentence: “Recent studies have identified, for example, the presence of human DNA in water samples, opening up new possibilities for the use of this technology (Goray *et al.*, 2024)”

Ecological and Restorative Justice as Guiding Principles

- This section focuses on the application of ecological justice within the Green Criminology framework through Recognition, Responsibility and Restoration
  - Recognition being the acknowledgment that damage to an ecosystem is a fundamental injustice
  - Responsibility referring to restructuring systems to recognise these fundamental injustices as punishable by law
  - And finally placing environmental restoration as a priority  in the legal ramifications of harm against nature.

Making the Invisible, Visible; molecular evidence of ecological damage

- Argues that eDNA, and other molecular genetic techniques allow a clear, causal link to be established between anthropogenic activities and environmental harm.

Systemic and Interdisciplinary understanding of environmental harm

- Using biological health indicators and metrics as measures for urgency of legal actions

Conclusions

- Focusing on interdisciplinary actions, bringing together science and green criminology for action
- Focus on community driven ecological justice

If applicable, is the statistical analysis and its interpretation appropriate?

Not applicable

Is the study design appropriate and is the work technically sound?

Yes

Is the work clearly and accurately presented and does it engage with the current literature?

Yes

Are the conclusions drawn adequately supported by the results?

Partly

Are sufficient details of methods and analysis provided to allow replication by others?

Not applicable

Are all the source data and materials underlying the results available?

No source data required

Reviewer Expertise:

eDNA, Molecular genetics

I confirm that I have read this submission and believe that I have an appropriate level of expertise to confirm that it is of an acceptable scientific standard, however I have significant reservations, as outlined above.

# Response

**Published:** 2025-10-10

**Authors:** Esteban Morelle-Hungría

**Affiliations:** Public Law, Universitat Jaume I, Castellón de la Plana, Valencian Community, Spain

We have carefully revised the manuscript following the reviewer’s insightful comments. All suggested references have been incorporated throughout the text and the reference list. We verified the accuracy of each citation and ensured conceptual consistency between green criminology, molecular genetics, and ecological justice. Additionally, we have strengthened the discussion with updated sources (e.g., Acharya-Patel et al., 2025; Lewis et al., 2024) highlighting the recent applications of molecular genetics to environmental monitoring. These additions enhance the paper’s methodological robustness and interdisciplinary depth. We sincerely thank the reviewer for their valuable feedback, which has significantly improved the theoretical and empirical foundations of our work.
